# Short-term diagnostic stability of probable headache disorders based on the International Classification of Headache Disorders, 3rd edition beta version, in first-visit patients: a multicenter follow-up study

**DOI:** 10.1186/s10194-016-0605-1

**Published:** 2016-02-19

**Authors:** Byung-Su Kim, Heui-Soo Moon, Jong-Hee Sohn, Myong-Jin Cha, Tae-Jin Song, Jae-Moon Kim, Jeong Wook Park, Kwang-Yeol Park, Soo-Jin Cho, Soo-Kyoung Kim

**Affiliations:** Department of Neurology, Daejin Medical Center, Bundang Jesaeng General Hospital, Seongnam, South Korea; Department of Neurology, Kangbuk Samsung Hospital, Sungkyunkwan University School of Medicine, Seoul, South Korea; Department of neurology, Chuncheon Sacred Heart Hospital, Hallym University College of Medicine, Chuncheon-si, South Korea; Department of neurology, National Police Hospital, Seoul, South Korea; Department of Neurology, Ewha Womans University School of Medicine, Seoul, South Korea; Department of Neurology, Chungnam National University College of Medicine, Daejeon, South Korea; Department of Neurology, Uijeongbu St.Mary’s Hospital, The Catholic University of Korea College of Medicine, Uijeongbu, South Korea; Department of Neurology, Chung-Ang University Hospital, Chung-Ang University College of Medicine, Seoul, South Korea; Department of neurology, Dongtan Sacred Heart Hospital, Hallym University College of Medicine, 40, Seokwoo-dong, Hwaseong-si, Gyeonggi-do 445-170 South Korea; Department of neurology, Gyeongsang National University School of Medicine, Jinju, South Korea

**Keywords:** Headache, Migraine, Tension-type headache, Tracking, Stability, Probable diagnosis

## Abstract

**Background:**

A “Probable headache disorder” is diagnosed when a patient’s headache fulfills all but one criterion of a headache disorder in the 3rd beta edition of the International Classification of Headache Disorder (ICHD-3β). We investigated diagnostic changes in probable headache disorders in first-visit patients after at least 3 months of follow-up.

**Methods:**

This was a longitudinal study using a prospective headache registry from nine headache clinics of referral hospitals. The diagnostic change of probable headache disorders at baseline was assessed at least 3 months after the initial visit using ICHD-3β.

**Results:**

Of 216 patients with probable headache disorders at baseline, the initial probable diagnosis remained unchanged for 162 (75.0 %) patients, while it progressed to a definite diagnosis within the same headache subtype for 45 (20.8 %) by fulfilling the criteria during a median follow-up period of 6.5 months. Significant difference on the proportions of constant diagnosis was not found between headache subtypes (*P* < 0.935): 75.9 % for probable migraine, 73.7 % for probable tension-type headache (TTH), and 76.0 % for probable other primary headache disorders (OPHD). Among patients with headache recurrence, the proportion of constant diagnosis was higher for probable migraine than for probable TTH plus probable OPHD (59.2 vs. 23.1 %; *P* < 0.001). The proportions of constant diagnosis did not significantly differ by follow-up duration (>3 and ≤ 6 months vs. > 6 and ≤ 10 months) in probable migraine, probable TTH, and probable OPHD, respectively.

**Conclusions:**

In this study, a probable headache diagnosis, based on ICHD-3β, remained in approximately three-quarters of the outpatients; however, diagnostic stability could differ by headache recurrence and subtype. Probable headache management might have to consider these differences.

## Background

The International Classification of Headache Disorder (ICHD) defines a probable headache diagnosis as headache attacks fulfilling all but one of the diagnostic criteria for a definite headache diagnosis [1]. Because the ICHD stipulates that the essential diagnostic criteria of migraine and many other headache disorders are based mainly on clinical features, with no decisive pathological or radiological finding, the concept of a probable diagnosis seems to be indispensable in making a headache diagnosis. In this regard, the application of probable diagnostic entities has the merit of reducing the proportion of unclassified headache diagnoses.

The diagnosis of probable headache disorders can be made in conditions of incomplete or atypical presentations of definite headache attacks [2]. In the real world, and in both epidemiological and clinical fields, it is certain that some headache attacks do not fully meet the definite diagnostic criteria of the ICHD, and the reported prevalence of probable headache disorder is considerable [3–8]. In the ICHD 2nd edition, there were nine probable diagnostic entities, including three for migraine, three for tension-type headache (TTH), and three for trigeminal autonomic cephalalgias (TAC). In the ICHD, 3rd edition beta version (ICHD-3β), the diagnostic entities have been expanded because of the addition of probable or probably criteria used for other primary headache disorders (OPHD) and reversible cerebral vasoconstriction syndrome (RCVS) [1]. Thus, a probable diagnosis is possible in most diagnostic categories in primary headache disorders, with a few exceptions, such as chronic migraine and primary thunderclap headache. Although diagnostic changes in probable diagnoses over time still remain to be determined, diagnostic stability might be valuable information for clinicians, in terms of treatment planning and prognostic predictions [8–10].

Therefore, the aim of this study was to investigate diagnostic changes and the stability of probable headache disorders in first-visit patients after a follow-up of at least 3 months. We evaluated diagnostic consequences by baseline characteristics and missing criteria for a definite diagnosis in each headache subtype.

## Methods

### Study design and subjects

This was a substudy of the headache registry using ICHD-3β for the first-visit patients (HEREIN) study using prospectively collected multicenter data obtained from consecutive first-visit headache outpatients in the headache clinics of 11 hospitals in Korea between August 2014 and February 2015 [11–13]. The study design of the HEREIN study was proposed and reviewed at regular educational meeting of the Korean Headache Society after a request regarding the application of ICHD-3β; study members were board-certified neurologists with special interests in the headache field. Consecutive patients visiting outpatient headache clinics for the first time were enrolled by 11 neurologists, of whom 1 managed the entire dataset. The patients had headache as their chief complaint in their outpatient visit. They ranged from 19 to 100 years of age and were Koreans with no disability in communication relevant to appropriate history-taking. Exclusion criteria were the presence of other chief complaints besides headache, not of Korean ethnicity, significant communication disabilities due to hearing, speech, or cognition impairments, and any other serious medical or psychiatric problem according to the physician’s judgment. The classification of headache disorder was conducted within the current headache phenotypes using ICHD-3β by each investigator, based on the initial evaluation including the structured questionnaire, clinical evaluation, and laboratory or neuroimaging studies, as needed. For the analysis, the investigator selected the most important headache for each patient.

Nine hospitals from the HEREIN study joined this longitudinal substudy to assess the diagnostic stability of probable headache disorders. This study was conducted prospectively in five tertiary and two secondary referral university hospitals and two secondary referral general hospitals throughout Korea (Seoul, 3; Daejeon, 1; Gyeonggi-do, 3; Gangwon-do, 1; Gyeongsangnam-do, 1) according to the Declaration of Helsinki and Good Clinical Practices. The criteria for study eligibility were as follows: 1) patients with a probable or probably headache diagnosis in the HEREIN study, and 2) agreement to participate in the study. The study protocol and informed consent or a use-of-information agreement form were reviewed and approved by the institutional review board (IRB) of each hospital. Each patient gave written informed consent prior to their participation in the study or waived the informed consent process, according to the decision of the IRB of each hospital.

### Follow-up assessment

Among 1414 first-visit headache patients from the nine hospitals, 276 patients had probable or probably headache diagnoses at baseline (270 probable diagnoses and 6 probably RCVS diagnoses). The researchers contacted those patients after a follow-up of 3 or more months. If the subjects visited the outpatient headache clinics continuously, researchers asked about participation in the study in the clinical setting. Also, if the subjects missed a follow-up visit, they were asked about their intent to participate in this study prior to starting a telephone interview. During face-to-face or telephone interviews, we collected data on recurrence of headaches, similarity of headache characteristics between baseline and additional attacks, clinical course, and response to headache treatment. We further investigated any available results of diagnostic work-ups that were also performed on the study subjects during the follow-up period. Based on these data, we decided on the diagnostic stability of the probable headache disorders in ICHD-3β, and conversion from a probable to a definite headache diagnosis. If the headache profile of the study subjects did not fully meet the conditions of a definite headache diagnosis, they were considered to have a constant probable diagnosis after the 3-month follow-up.

### Statistical analysis

In descriptive statistics, continuous variables are presented as means ± standard deviation (or medians with interquartile range), and were evaluated statistically using the Student’s *t*-test. Categorical variables are reported as numbers (percentages), and the test statistic was based on the *χ*^2^ test or Fisher’s exact test. Due to the small sample size of probable TAC and probably RCVS groups, we included probable migraine, probable TTH, and probable OPHD groups in statistical comparison of diagnostic stability between headache subtypes. If cell number of OPHD is not enough to conduct the categorical statistical analyses, we compared diagnostic stability between probable migraine vs. probable TTH plus probable OPHD groups. To evaluate the diagnostic stability over time, we compared the proportions of constant headache diagnosis between subgroups of follow-up duration (>3 and ≤ 6 months vs. > 6 and ≤ 10 months) in each headache subtype. All statistical analyses were conducted with SPSS for Windows software (ver. 18.0; SPSS Inc., Chicago, IL. USA). All reported *P*-values are two-tailed, and those < 0.05 were considered to indicate statistical significance.

## Results

### Study subjects

**Fig. 1:**
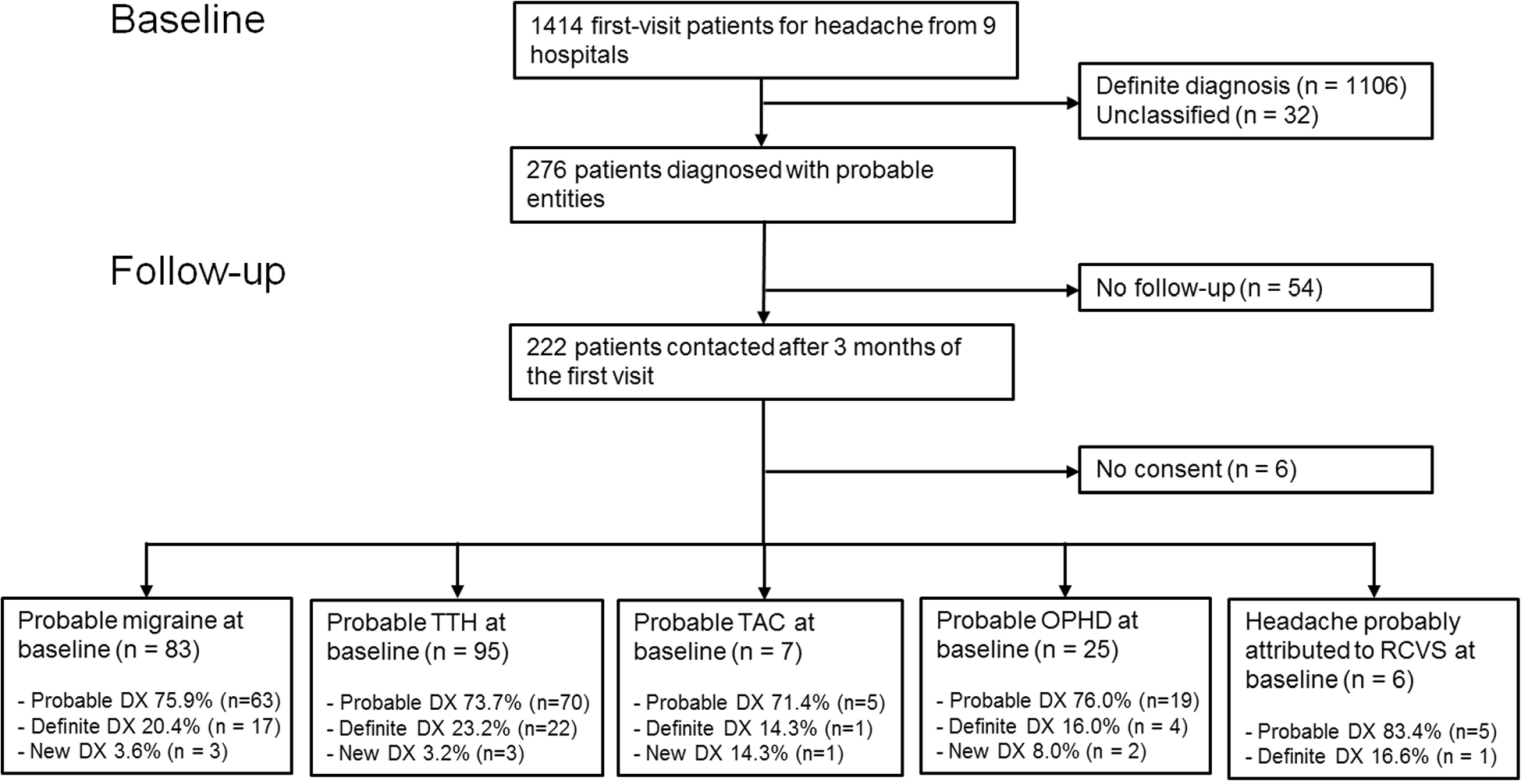
Flow chart indicating the participation and follow-up of the study patients (abbreviations: TTH, tension-type headache; TAC, trigeminal autonomic cephalalgias; OPHD, other primary headache disorders; RCVS, reversible cerebral vasoconstriction syndrome)

In total, 276 patients with probable or probably headache disorders were selected for the present study among 1414 first-visit headache patients from the nine hospitals (Fig. 1). From March 2015 to June 2015, we contacted 276 patients with probable or probably headache diagnoses: 54 were lost to follow-up and 6 refused to participate in the study. There was no difference in age (47.5 vs. 46.9 years) or gender (54.0 vs. 57.1 % female) between the 276 patients from the nine hospitals and 35 patients from the remaining two hospitals. Finally, 216 patients (mean age: 47.7 ± 13.5 years, 113 females) were included in the study analysis. The numbers by headache subtype were as follows: 83 (38.4 %) with probable migraine, 95 (44.0 %) with probable TTH, 7 (3.2 %) with probable TAC, 25 (11.6 %) with probable OPHD, and 6 (2.8 %) with headache probably attributed to RCVS. In the 216 patients, age was not significantly different (47.7 vs. 37.7 years), but the proportion of females was higher (52.3 vs. 33.3 %), compared with the 6 patients refusing to participate. The baseline characteristics of the study subjects are shown in Table 1.

**Fig. 2:**
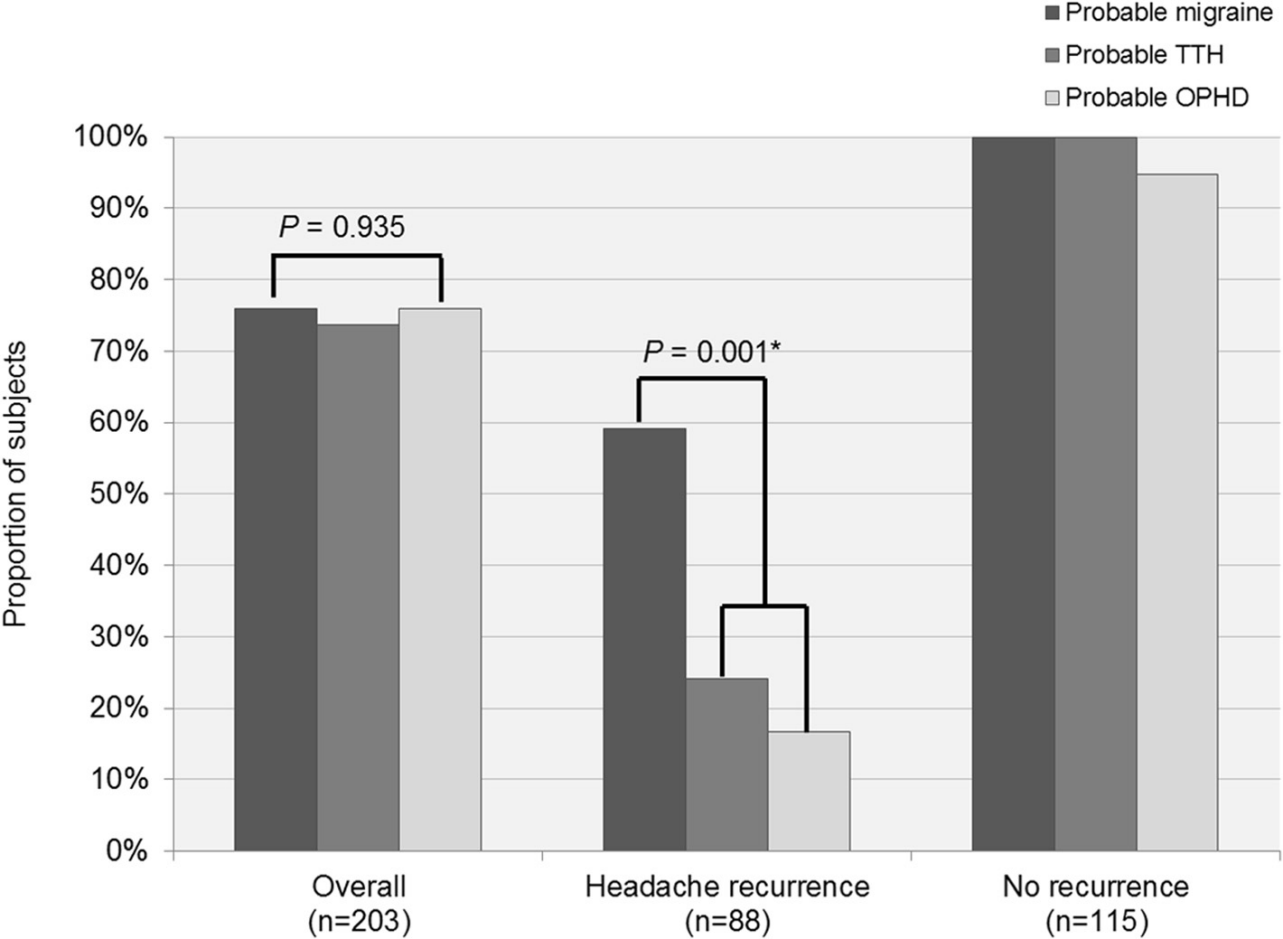
The proportions of whom have constant probable diagnosis between probable migraine, probable tension-type headache, and probable other primary headache disorders according to headache recurrence during follow-up (abbreviations: TTH, tension-type headache; OPHD, other primary headache disorders; FU, follow-up). *Comparison between probable migraine versus probable TTH plus probable OPHD groups

**Table 1:** Baseline characteristics of study patients

Characteristics	*n* = 216
Age, years	47.7 ± 13.5
Female	113 (52.3)
Headache intensity (0–10 VAS)	5.9 ± 2.2
Duration of current headache bout, months	0.8 (0.2–3.0)
Onset age, years	45.7 ± 14.2
Headache subtype	
Probable migraine	83 (38.4)
Probable TTH	95 (44.0)
Probable TAC	7 (3.2)
Probable OPHD	25 (11.6)
Headache probably attributed to RCVS	6 (2.8)

Data are presented as means ± standard deviation, medians with interquartile range or as numbers (%)

*Abbreviations*: *VAS* visual analogue scale, *TTH* tension-type headache, *TAC* trigeminal autonomic cephalalgias, *OPHD* other primary headache disorders, *RCVS* reversible cerebral vasoconstriction syndrome

### Follow-up

During a median follow-up period of 6.5 months (range: 4.0–10.0 months), 91 (41.2 %) patients had additional headache attacks. The proportions regarding the recurrence of headache attacks by primary headache subtype were as follows: 59.0 % (*n* = 49) for probable migraine, 34.7 % (*n* = 33) for probable TTH, 42.9 % (*n* = 3) for probable TAC, and 24.0 % (*n* = 6) for probable OPHD; the proportion of the headache recurrence was significantly higher for migraine than for non-migraine primary headache (59.0 vs. 33.1 %; *P* < 0.001). There was no additional headache attack in the patients with headache probably attributed to RCVS.

Of the 91 patients, only 8 (3.7 %) experienced another headache distinct from the headache at baseline, of which 5 were diagnosed with new definite diagnoses, whereas the other 3 had both the previous probable diagnosis and another new probable diagnosis. Of 83 patients suffering a similar headache during the follow-up period, the initial probable diagnosis was changed to a definite headache diagnosis for 43 patients, while it was maintained for the other 40 patients. One patient (initial diagnosis: 3.5.2 probable paroxysmal hemicranias) was diagnosed as 14.2 headache unspecified, because the headache treatment response during the follow-up differed from the first one.

Of 125 patients without recurrence of headache, most (*n* = 123, 98.4 %) were given a constant probable diagnosis, while an initial probable diagnosis was changed to a definite diagnosis for the other two patients: for one with primary stabbing headache, additional medical history obtained during the follow-up period satisfied the full diagnostic criteria and for the other with headache probably attributed to RCVS, cerebral vasoconstriction was found in follow-up cerebral angiography.

Of the 216 patients, 45 (20.8 %) eventually had a definite diagnosis within the same headache subtype, because of fulfilling the criteria during the follow-up period (Fig. 1). A probable diagnosis was more likely to progress to a definite diagnosis of the same headache subtype for patients with headache recurrence than for those without (47.3 vs. 1.6 %; *P* < 0.001).

### Diagnostic stability

The initial probable diagnosis remained unchanged for 162 (75.0 %) patients. The proportions of constant probable diagnosis by primary headache subtype were as follows: 75.9 % (*n* = 63) for probable migraine, 73.7 % (*n* = 70) for probable TTH, 71.4 % (*n* = 5) for probable TAC, and 76.0 % (*n* = 19) for probable OPHD (Fig. 1). The proportions of constant probable diagnosis were not significantly different according to the baseline characteristics of age (≥50 vs. < 50 years), gender (female vs. male), headache intensity, defined by VAS (≥7 vs. < 7), headache onset age (≥50 vs. < 50 years), and headache duration (≥1 vs. < 1 month; data not shown).

Regarding the constant probable diagnosis, no significant difference was observed between probable migraine, probable TTH, and probable OPHD (*P* = 0.935); however, subgroup analysis involving patients with headache recurrence showed that the proportion of constant diagnosis was significantly higher for probable migraine group and probable TTH plus probable OPHD group (*P* = 0.001, Fig. 2). Of the six patients with headache probably attributed to RCVS, the probable diagnosis was maintained for five (83.3 %) patients.

**Fig. 3:**
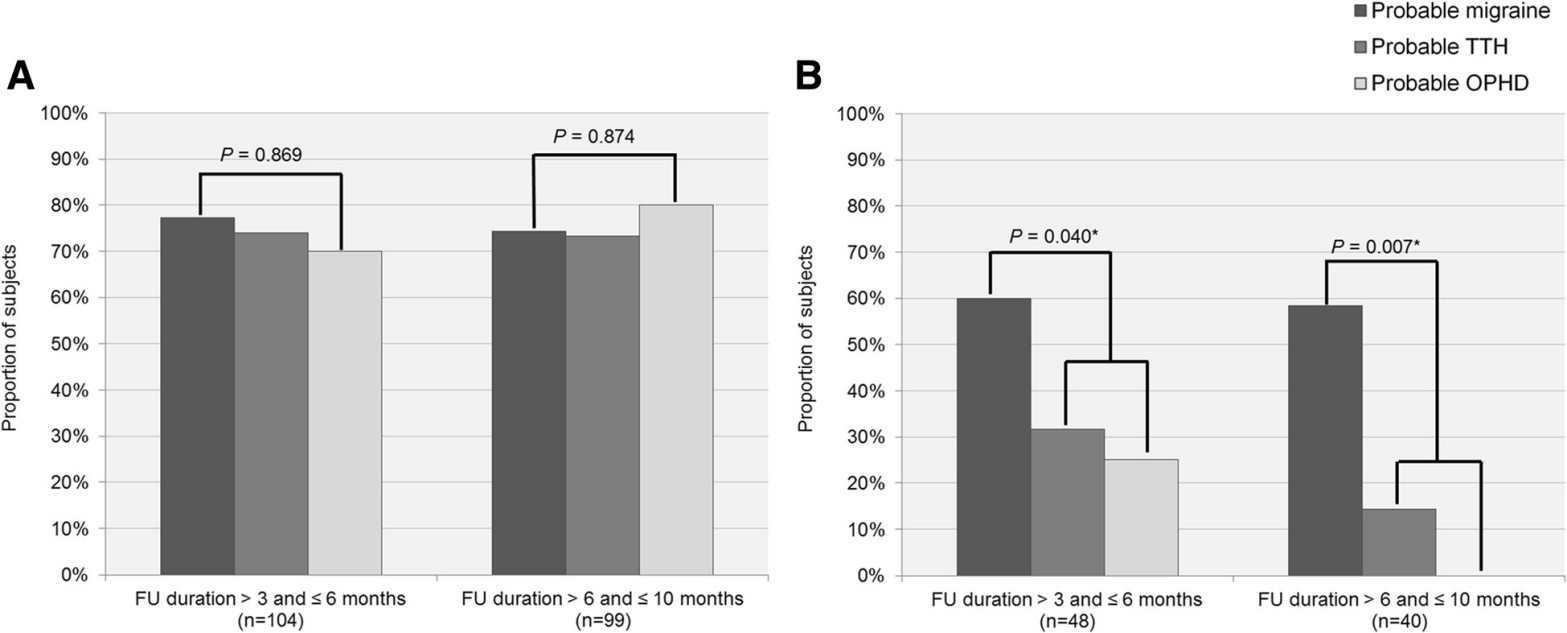
The proportions of whom have constant probable diagnosis between probable migraine, probable tension-type headache, and probable other primary headache disorders according to follow-up duration (>3 and ≤ 6 months vs. > 6 and ≤ 10 months) among (**a**) overall patients and (**b**) patients with headache recurrence (abbreviations: TTH, tension-type headache; OPHD, other primary headache disorders; FU, follow-up). *Comparison between probable migraine versus probable TTH plus probable OPHD groups

The proportions of constant diagnosis between probable migraine, probable TTH, and probable OPHD were compared in 2 subgroups defined by follow-up duration (>3 and ≤ 6 months vs. > 6 and ≤ 10 month), respectively (Fig. 3). Among overall patients, there was no difference on the proportions of constant diagnosis between the 3 headache subtypes in both the subgroups of follow-up period (*P* = 0.869 and *P* = 0.874, respectively). In patients with headache recurrence, the proportion of constant diagnosis was significantly higher for probable migraine group than for probable TTH plus probable OPHD group in both the subgroups of follow-up period (*P* = 0.040 and *P* = 0.007, respectively).

We further compared the proportions of constant diagnosis by follow-up duration (>3 and ≤ 6 months vs. > 6 and ≤ 10 months) in probable migraine, probable TTH, and probable OPHD, respectively. The proportions of constant diagnosis were not significantly different according to the follow-up duration in all subtypes: 77.2 vs. 73.3 % for probable migraine (*P* = 0.757), 74.0 vs. 73.3 % for probable TTH (*P* = 0.941), and 70.0 vs. 80.0 % for probable OPHD (*P* = 0.653). Among the patients with headache recurrence, the proportions of constant diagnosis according to follow-up duration were similar in probable migraine (60.0 vs. 58.3 %; *P* = 0.906). The proportion of constant probable TTH was lower in group with follow-up duration > 6 and ≤ 10 months; the difference was not statistically significant (31.6 vs. 14.3 %; *P* = 0.416). Of 4 OPHD patients followed for 3–6 months, only 1 patient (25.0 %) had constant probable diagnosis, whereas probable diagnosis of all 2 patients followed for > 6 and ≤ 10 months progressed to definite diagnosis.

The proportions of diagnostic changes by missing diagnostic criteria in each probable diagnostic entity are presented in Table 2. The number of attacks was the most common missing criterion in both probable migraine and probable TTH. In probable TTH, the proportion of constant probable diagnosis was lowest in patients with the missing criterion of total headache period. In OPHD, 14 patients were given an initial diagnosis of probable new daily persistent headache (NDPH), because their persistent headache did not last for 3 or more months at baseline. The probable NDPH diagnosis was not changed for most of them (*n* = 13, 92.9 %), because their headache did not last for more than 3 months during follow-up. The other patient had a new definite headache diagnosis (primary stabbing headache) during follow-up.

**Table 2:** Proportions of diagnostic changes after the 3-month follow-up according to missing criterion of headache subtype at baseline

Missing criterion	No. of cases	Follow-up diagnosis		
		Probable diagnosis	Definite diagnosis	New diagnosis or headache unspecified
Probable migraine				
Number of attacks	33	23 (69.7)	8 (24.2)	2 (6.0)
Duration of attack	15	13 (86.7)	2 (13.3)	0 (0.0)
Features of headache	6	6 (100.0)	0 (0.0)	0 (0.0)
Accompanying symptoms	28	20 (71.4)	7 (25.0)	1 (3.6)
Characteristics of aura	1	1 (100.0)	0 (0.0)	0 (0.0)
Probable TTH				
Number of attacks	66	49 (74.2)	15 (22.7)	2 (3.0)
Duration of attack	10	10 (100.0)	0 (0.0)	0 (0.0)
Features of headache	8	6 (75.0)	1 (12.5)	1 (12.5)
Total headache period	11	5 (45.5)	6 (54.5)	0 (0.0)
Probable TAC				
Number of attacks	1	1 (100.0)	0 (0.0)	0 (0.0)
Duration of attack	1	1 (100.0)	0 (0.0)	0 (0.0)
Accompanying symptoms	1	0 (0.0)	0 (0.0)	1 (100.0)^a^
Headache frequency	1	1 (100.0)	0 (0.0)	0 (0.0)
Response to treatment	1	1 (100.0)	0 (0.0)	0 (0.0)
Headache location	1	0 (0.0)	1 (100.0)	0 (0.0)
Total headache period	1	1 (100.0)	0 (0.0)	0 (0.0)
Probable OPHD				
Number of attacks	7	4 (57.1)	2 (28.6)	1 (14.3)
Duration of attack	2	1 (50.0)	1 (50.0)	0 (0.0)
Accompanying symptoms	1	1 (100.0)	0 (0.0)	0 (0.0)
Total headache period	14	13 (92.9)	0 (0.0)	1 (7.1)
Characteristics of nummular headache	1	0 (0.0)	1 (100.0)	0 (0.0)
Headache probably attributed to RCVS				
Cerebral vasoconstriction	6	5 (83.3)	1 (16.7)	0 (0.0)

Data are presented as numbers (%)

*Abbreviations*: *TTH* tension-type headache, *TAC* trigeminal autonomic cephalalgias, *OPHD* other primary headache disorders, *RCVS* reversible cerebral vasoconstriction syndrome

^a^Headache unspecified

## Discussion

This study demonstrated that a probable diagnosis using ICHD-3β was maintained for three-quarters of first-visit headache patients during a median follow-up period of 6.5 months. Previously, a few studies reported the diagnostic stability of migraine and/or TTH; however, our study investigated the diagnostic stability of all the probable diagnoses listed in ICHD-3β using a multicenter headache registry. Among the overall patients, the stability of a probable diagnosis did not differ significantly according to headache subtype. However, diagnostic stability differed by headache subtype; probable migraine was more solid headache diagnosis than other headache subtypes, regardless of follow-up duration. In addition, we compared the diagnostic stability by follow-up duration in each headache subtype (probable migraine, probable TTH, and probable OPHD). The proportions of constant probable diagnosis did not significantly differ between follow-up duration periods (>3 and ≤ 6 months vs. > 6 and ≤ 10 months) in all headache subtypes. Considering that probable OPHD were new diagnostic entities in ICHD-3β, these diagnoses might be expected to be used widely. In terms of missing criteria, the diagnostic stability was relatively lower in the subgroups lacking data on time-based criteria, number of attacks, or total headache period – in migraine and TTH – because the proportion progressing to a definite diagnosis for these subgroups was higher than for other subgroups.

A headache diagnosis made at a first visit could change for various reasons in the process of follow-up, because a headache diagnosis is based on clinical characteristics and a time-based history [4, 9, 14–16]. In this regard, the diagnostic stability of probable diagnosis in headache outpatients may be considered to be less solid, because many clinicians generally give a probable diagnosis when the clinical characteristics or time-based history do not meet the diagnostic criteria of ICHD-3β [3, 4, 14]. However, in our study, the diagnostic stability of a probable diagnosis at the first visit seems to be substantial, whereas the proportions of progression to a definite diagnosis or a new diagnosis (or unspecified headache) were 20.8 and 4.2 %, respectively, during follow-up.

There have been few previous studies reporting diagnostic changes of probable diagnoses over time [3, 4]. Previous studies revealed the lower stability of a probable diagnosis compared with a definite diagnosis. The reported frequency for a constant diagnosis was 38.8–44.7 % for probable migraine and 24.6–53.9 % for probable TTH. Those findings differ from our study. The shorter follow-up duration (median: 6.5 months) of our study might be responsible for the higher diagnostic stability, because follow-up durations were 7 months for a recent adolescent study and 2.2 years for an epidemiological study. Nevertheless, the difference may not be easy to explain, because the demographic and study settings also differ between our and previous studies. Thus, additional studies in a similar setting are needed to confirm our findings.

An interesting finding in the present study was that patients with probable migraine were twice as likely to experience recurrence of headache compared with those with non-migraine primary headaches. Nonetheless, the diagnostic stability of probable migraine was not significantly different from those of other primary headache subtypes. Furthermore, subgroup involving the patients with headache recurrence showed that 59.2 % had constant probable migraine. These results indicate that probable migraine may be a more solid diagnosis than our expectations and considered to be migraine spectrum rather than simply an incomplete diagnosis [10, 17]. In addition, given the probability of headache recurrence in the near future, proper therapeutic management should be provided to first-visit probable migraine patients, because subjects with probable migraine usually suffer from comparable headache-related disability in comparison with definite migraine [5, 6, 8, 10, 18].

In the clinical setting, many TTH patients with number of attacks < 10 or total period < 3 months may come to a headache clinic first. They would be diagnosed with probable TTH under ICHD-3β. In the HEREIN study, probable TTH was the third most common headache subtype after migraine without aura and primary stabbing headache [11]. Most probable TTH cases (78.9 %) did not satisfy the criteria for number of attacks or total period at baseline, and their diagnostic stability was lower compared with subgroups of other missing criteria. Thus, to reduce the frequency of such probable TTH, there may be a need for more practical diagnostic criteria for TTH at a first visit.

Probable NDPH accounted for 56.0 % of probable OPHD in the study. Secondary causes that could mimic NDPH were not found over time [19, 20]. The persistent headaches ended within the follow-up period. Given that it may sometimes be challenging to distinguish NDPH from other primary headaches, further studies focusing on probable NDPH need to be conducted before the publication of ICHD-3 [1, 19, 20].

The major strengths of this study include its prospective design and the participation of headache specialists at multicenter headache clinics of referral tertiary and general hospitals. However, there are also several limitations. First, the study has limited generalizability, because clinic-visit patients were enrolled. Second, probable headache could be heterogeneous in terms of missing criteria; however, we could not conduct a more detailed subgroup analysis due to the sample size of our study patients. Third, the median follow-up duration was 6.5 months, which might not be enough to conclude the diagnostic consequences of probable headache disorders. Diagnostic stability could also be decreased and the possibility of the occurrence of new headache increased over a longer follow-up time [3, 9, 14]. Thus, these results should be interpreted with caution, and long-term follow-up studies are needed to confirm the findings. Finally, we did not use a headache diary or written report of headache attack to make a headache diagnosis. This could increase the risk of recall bias in the study.

## Conclusions

The present study showed that a probable headache diagnosis, based on ICHD-3β, remained in approximately three-quarters of the first-visit outpatients within the 10-month period. However, diagnostic stability could differ by headache recurrence and subtype. Probable headache management might have to consider these differences. Given the prevalence of probable headache, probable diagnoses could be deserved to be studied more closely and over the long term.

## Abbreviations

HEREIN: headache registry using the ICHD-3β for the first-visit patients
ICHD: international classification of headache disorders
ICHD-3β: International Classification of Headache Disorders, 3rd edition beta version
IRB: Institutional Review Board
NDPH: new daily persistent headache
OPHD: other primary headache disorders
RCVS: reversible cerebral vasoconstriction syndrome
TAC: trigeminal autonomic cephalalgias
TTH: tension-type headache
